# Temporary Transvenous Pacemaker Lead-Induced Cardiac Perforation Incidentally Detected by Right Ventriculography Prior to Leadless Pacemaker Implantation

**DOI:** 10.7759/cureus.84136

**Published:** 2025-05-14

**Authors:** Kazunori Omote, Tadao Aikawa, Yuki Ishidoya, Daisuke Sunaga, Naohiro Funayama

**Affiliations:** 1 Department of Cardiology, Hokkaido Cardiovascular Hospital, Sapporo, JPN; 2 Department of Cardiovascular Biology and Medicine, Juntendo University Graduate School of Medicine, Tokyo, JPN

**Keywords:** complication, leadless pacemaker, right ventricular perforation, right ventriculography, temporary transvenous pacemaker lead

## Abstract

Temporary transvenous pacemakers (TTPs) are lifesaving device in patients with hemodynamically unstable bradycardia, but are associated with serious complications, including right ventricular (RV) perforation. We present a case of a 91-year-old woman with complete atrioventricular block and bradycardia who received a TTP. After that, she underwent leadless pacemaker implantation. Preprocedural right ventriculography (RVG) revealed an incidental RV wall perforation by the TTP lead. After preparing for pericardiocentesis, the lead was safely extracted without complications. This case highlights the utility of RVG in detecting lead-related complications, where it is necessary to prepare pericardiocentesis prior to lead removal.

## Introduction

A temporary transvenous pacemaker (TTP) is an essential intervention for managing hemodynamically unstable bradycardia. Stabilizing cardiac rhythm supports circulatory function and facilitates adequate oxygen transport to peripheral tissues. Nevertheless, its invasive nature is accompanied by a certain incidence of procedure-related complications and is associated with worse clinical outcomes [1].

Among these complications, the right ventricular (RV) perforation caused by a TTP lead is observed in less than 1% of cases [1], which is a potentially fatal complication that can need surgical treatment. The lead-induced perforation is typically detected by computed tomography (CT), chest X-ray, or the pericardial effusion via echocardiography [2,3]. Here, we present a unique case of temporary pacemaker lead perforation, incidentally detected by right ventriculography (RVG) prior to leadless pacemaker implantation.

## Case presentation

**Figure 1:**
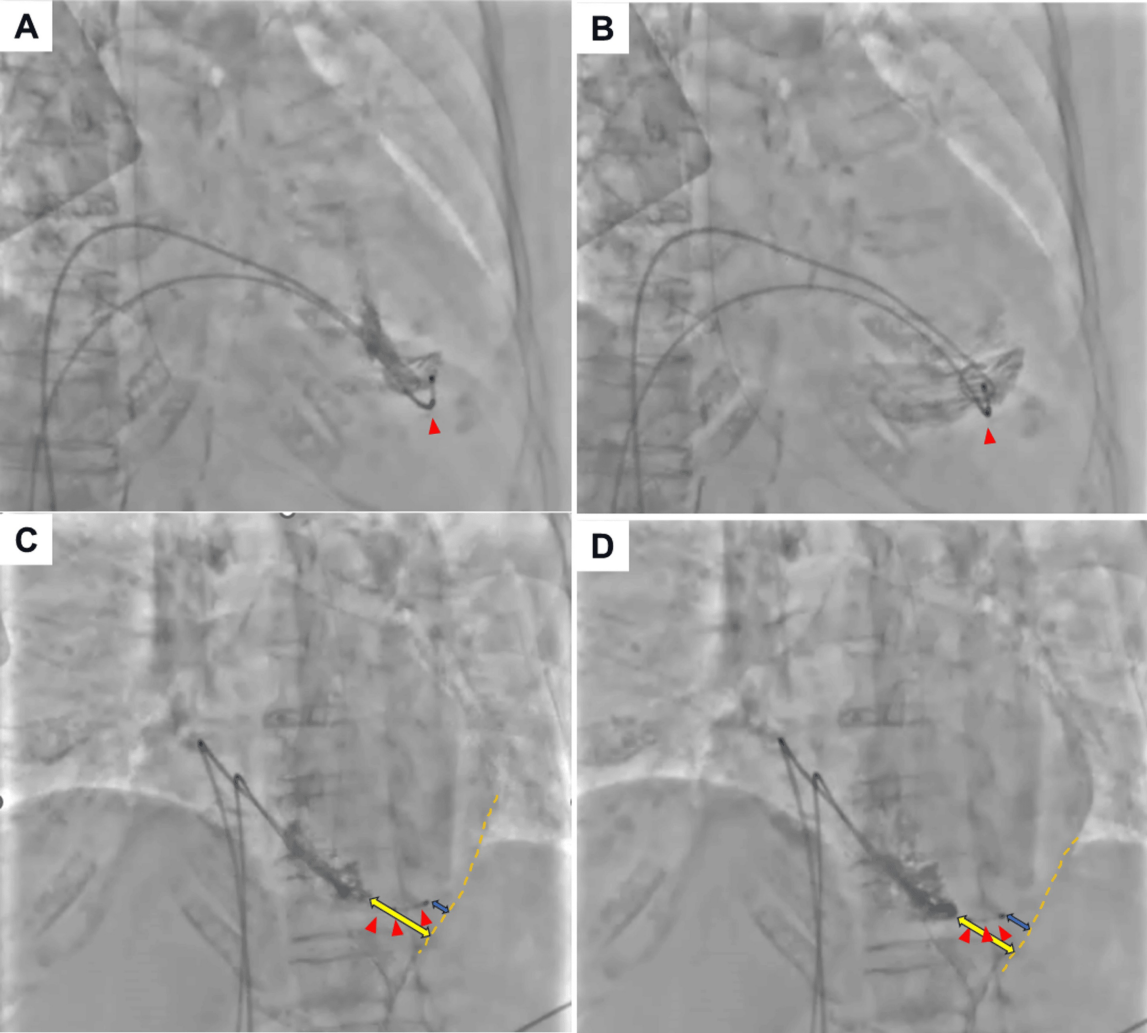
Temporary transvenous pacemaker lead-induced cardiac perforation detected by right ventriculography. The tip of the temporary transvenous pacemaker lead is sticking out of the right ventricle chamber. Right anterior oblique at systole (A), right anterior oblique at diastole (B), left anterior oblique at systole (C), and left anterior oblique at diastole (D). The red arrow indicates the tip of the temporary transvenous pacemaker lead extending from the right ventricle. The yellow bidirectional arrow represents the distance from the right ventricular apex to the epicardium. The blue bidirectional arrow shows the distance from the temporary transvenous pacemaker tip to the epicardium.

A 91-year-old woman with a history of severe aortic stenosis and heart failure presented to our emergency department with complaints of nausea and fatigue. On examination, her heart rate was 30 bpm and blood pressure was 82/50 mmHg. Electrocardiography showed complete atrioventricular block. A temporary transvenous pacemaker (TTP) was urgently inserted via the right internal jugular vein to improve her symptoms. Shortly thereafter, as the patient suffered a cardiogenic embolic stroke, she needed to receive treatment by the department of neurosurgery. During her prolonged hospitalization, the TTP lead was repositioned via the left femoral vein due to concerns regarding infection. After clinical stabilization, a leadless pacemaker implantation was planned. Preprocedural RVG that was performed to assess right ventricular anatomy, unexpectedly revealed that the tip of the temporary pacing lead had perforated the RV free wall (Figure 1, Videos 1, 2).

**Video 1:** Right ventriculography at right anterior oblique view.

**Video 2:** Right ventriculography at left anterior oblique view.

Given the risk of cardiac tamponade during lead removal, we prepared for pericardiocentesis. The lead was subsequently extracted without complication. The patient remained stable postoperatively, with no signs of pericardial effusion or hemodynamic compromise.

## Discussion

TTPs are essential in the management of bradycardia. However, despite their widespread use, complications such as myocardial perforation remain a serious concern. RV perforation is a rare but potentially life-threatening complication, reported in less than 1% of TTP insertions [1]. In this case, the RV perforation was incidentally identified during a routine RVG performed prior to a leadless pacemaker implantation. This is notable because RVG is not a standard modality for diagnosing TTP-related complications. Traditionally, echocardiography, CT, or chest radiographs can identify signs of perforation, such as pericardial effusion or mispositioned leads [2,3]. Our finding suggests that RVG is able to detect this rare complication.

Several risk factors may have contributed to the perforation in this elderly patient, including prolonged TTP use, multiple vascular access changes, and age-related myocardial fragility [4,5]. Advanced age is particularly significant, as tissue fragility and comorbid conditions increase the incidence of lead-related myocardial injury.

This case reinforces the need for careful surveillance and consideration of early conversion to permanent or leadless pacing in patients requiring prolonged temporary pacing. Moreover, preprocedural planning should include readiness for emergency interventions such as pericardiocentesis, especially when imaging suggests potential lead-related complications. As the use of leadless pacemakers is increasing, preprocedural anatomical imaging, such as RVG, may help detect silent perforations or malposition, potentially improving procedural safety [6].

## Conclusions

We experienced a unique case of a TTP lead-induced RV perforation incidentally detected by RVG prior to leadless pacemaker implantation. In this era of increasing use of leadless pacemaker devices, it is important to recognize the presence of this phenomenon. In such cases, it is necessary to prepare pericardiocentesis prior to TTP lead removal.

## References

[1] Metkus TS, Schulman SP, Marine JE, Eid SM (2019). Complications and outcomes of temporary transvenous pacing: an analysis of >360,000 patients from the National Inpatient Sample. Chest.

[2] Asano M, Mishima A, Ishii T, Takeuchi Y, Suzuki Y, Manabe T (1996). Surgical treatment for right ventricular perforation caused by transvenous pacing electrodes: a report of three cases. Surg Today.

[3] Erol MK, Sevimli S, Ates A (2005). Pericardial tamponade caused by transvenous temporary endocardial pacing. Heart.

[4] Kofune T, Watanabe I, Okubo K, Okumura Y, Masaki R, Shindo A, Saito S (2005). Effect of IKr blocker nifekalant on atrial action potential duration after successful internal cardioversion of chronic atrial fibrillation. Pacing Clin Electrophysiol.

[5] Mahapatra S, Bybee KA, Bunch TJ, Espinosa RE, Sinak LJ, McGoon MD, Hayes DL (2005). Incidence and predictors of cardiac perforation after permanent pacemaker placement. Heart Rhythm.

[6] Reddy VY, Exner DV, Cantillon DJ (2015). Percutaneous implantation of an entirely intracardiac leadless pacemaker. N Engl J Med.

